# Inguinal Hernias Represent the Most Frequent Surgical Complication after Kasai in Biliary Atresia Infants

**DOI:** 10.1155/2015/383791

**Published:** 2015-07-09

**Authors:** Omid Madadi-Sanjani, Nathalie Carl, Thomas Longerich, Claus Petersen, Julia H. K. Andruszkow

**Affiliations:** ^1^Center of Pediatric Surgery, Hannover Medical School, Carl-Neuberg-Strasse 1, 30625 Hannover, Germany; ^2^Institute of Pathology, RWTH Aachen University Hospital, Pauwelsstrasse 30, 52074 Aachen, Germany

## Abstract

Biliary atresia (BA) is an orphan medical condition of the newborn, resulting in end-stage liver cirrhosis due to obliterative cholangiopathy of the extrahepatic bile duct. Although Kasai's hepatoportoenterostomy (KPE) is the well-established first-line therapy, little is known about its surgical complications. 153 patients receiving open KPE treated at a single center between 1994 and 2014 were analysed retrospectively regarding short-term complications and survival with the native liver. In brief, 40.5% of patients suffered from 1–3 surgical complications, inguinal hernias (IH) being most prevalent (40.0%). In BA patients, incidence of IH was associated with male gender (*p* = 0.002), the syndromic form of BA (*p* = 0.038), and percutaneous drainage for ascites (*p* = 0.002). No association was found with prematurity (*p* = 0.074) or birth weight (*p* = 0.912) in our study. In conclusion, IH frequently develops after open KPE of BA patients, but this complication does not negatively affect the patient's outcome. Nevertheless, inspection of the internal inguinal ring and prophylactic closure of inapparent hernias should be discussed in order to prevent secondary surgical procedures.

## 1. Introduction

Biliary atresia (BA) is known as an orphan disease of the newborn, which manifests by progressive inflammation of the extrahepatic biliary tract [1, 2]. Without surgical intervention, children universally develop an obliterative cholangiopathy resulting in end-stage liver cirrhosis [1, 3]. To restore bile flow, Kasai's hepatoportoenterostomy (KPE) is performed in order to achieve survival with the patient's own liver [2, 3]. Nevertheless, due to progressive liver failure, 70–80% of patients require liver transplantation (LTx) on long-term perspective [2]. Although the sequential therapy of KPE and LTx improved survival rates of contemporary affected children approximately to 80–90%, both treatments still comprise potential comorbidities and complications [4]. In this respect, several studies described complications of LTx resulting in limited graft survival or increased mortality [5–7], while different analyses revealed long-term complications after KPE like splenomegaly, recurrent cholangitis, or portal hypertension [8–10]. However, little is known about surgical short-term complications after open KPE and their impact on the survival with the children's native liver. Zani and Davenport observed an increased incidence of inguinal hernias (IH) within the first month after KPE but failed to detect any independent risk factors [11]. The authors assumed that IH were related to the severity of liver damage and the extent of ascites as suggested by increased transaminases [11]. However, Joob and Wiwanitkit could not validate these findings in an independent larger study group [12].

Therefore, the present study aims at the identification of frequent short-term complications after open KPE and the validation of potential risk factors in BA patients. In particular, the present study focused on the incidence of IH in BA patients and its impact on survival with the children's native liver.

## 2. Materials and Methods

The present study follows the guidelines of the revised UN Declaration of Helsinki in 1975 and its latest amendment in 2008 (6th revision) and was approved by the Local Research Ethical Committee (number 41/2000). Informed consent was obtained from the patient's legal guardian before inclusion into the study.

### 2.1. Study Design and Inclusion Criterion

A retrospective analysis of patients with initial diagnosis of BA treated in the Center of Pediatric Surgery at the Hannover Medical School, Hannover, Germany, over a 20-year period (February 1994 to February 2014) was performed. Inclusion criterion was open KPE as surgical first-line therapy. Patients with incomplete data referring to loss of follow-up were excluded from the study.

### 2.2. Patients' Characteristics, Surgical Complications, and Outcome

Patients' gestational age, birth weight, gender, and the presence of syndromic BA were determined. Furthermore, patients' age at hospital admission and at the time of open KPE as well as procedural details (e.g., primary percutaneous drainage) was recorded. All surgical and interventional complications emerging after open KPE were analyzed. Primary outcome was defined as survival with the native liver until LTx or death. Time of follow-up until primary outcome was documented.

### 2.3. Statistical Analysis

The data were analyzed using the Statistical Package for the Social Sciences (SPSS, version 22 for windows, IBM Inc., Somers, NY, USA). Incidences were presented with counts and percentages while continuous values were presented as median with 25- and 75-interquartile ranges (IQR 25–75). Differences of categorical variables between the groups were evaluated with Fisher's exact test. The Spearman rank correlation coefficient was used to determine the association between IH and patients' characteristics (gender, syndromic BA, percutaneous drainage, and survival with the native liver).

In order to analyze the impact of patients' characteristics on the development of complications, a multivariate logistic regression analysis was performed with IH as target variable and age at KPE, gestational age, percutaneous drainage, and male gender, prematurity, syndromic BA, and birth weight as potential predictors. Odds ratios (OR) with 95% confidence intervals (95% CI) were noted.

Survival with the native liver during time of follow-up was analyzed with Kaplan-Meier survival plots using GraphPad Prism (version 6.00 for Windows, GraphPad Software, La Jolla, CA, USA). A comparison of survival curves between hernia patients and nonhernia patients was performed via Log-rank (Mantel-Cox) test. In general, a two-sided *p* value <0.05 was considered to be significant.

## 3. Results

### 3.1. Patient Characteristics

194 patients diagnosed with BA were treated by open KPE as surgical first-line therapy at the Center of Pediatric Surgery at Hannover Medical School between 1994 and 2014. A total of 41 patients (21.1%) were lost during follow-up.

Thus, 153 patients could be included. The median gestational age at birth was 39 weeks (IQR: 38–40 weeks) with a median birth weight of 3,270 grams (IQR: 2,830–3,540 grams). Twenty-three patients (15.0%) were prematurely born (e.g., prior to the 37th gestational week or less than 2,500 grams of body weight). The syndromic form of BA was diagnosed in 4.6% (*n* = 7) with the associated congenital anomalies listed in Table 1. Most children were female (56.2%, *n* = 86). The median age at referral was 53 days (IQR: 36.5–72.5 days). The median age at intervention was 56 days (IQR: 40.0–75.5 days). Percutaneous drainage was primarily performed during surgery in 51% of children (*n* = 78). The median follow-up time after KPE was 7.8 months (IQR: 4.8–17.1 months) and the median overall survival with the native liver was 10.2 months (IQR: 6.2–43.8 months).

### 3.2. Surgical Complications after Open KPE

40.5% (*n* = 62) of the study population developed a total of 75 surgical complications. The majority of children (85.5%, *n* = 53) suffered from one surgical complication while 8.1% (*n* = 5) developed two and 6.5% (*n* = 4) developed three complications. In general, complications of grade III or grade IV according to Dindo et al. [13] were found in most patients (95.2%, *n* = 59) while 3 patients (4.8%) died due to a lethal complication (Table 2). Two of these complications were related to small bowel-related comorbidities. The first child was diagnosed with an acute abdomen due to subtotal intestinal necrosis three months after open KPE. The second child suffered from postoperative adhesions five months after open Kasai and finally died from ileus. The third patient developed esophageal varices and died seven months after open KPE due to variceal hemorrhage. Taken together, our study revealed that lethal complications may develop within the first year after KPE and symptoms like sudden abdominal pain require for an alerted physician.

### 3.3. Inguinal Hernias as Most Frequent Complication

The most frequent surgical complication recorded after open KPE was IH (Table 2). The median interval between KPE and diagnosis of IH was 18 days (IQR: 8.0–45.5 days). Hernia repair was performed in all patients. In addition, one patient (3.3%) suffered from IH prior to KPE and received hernia repair during Kasai's procedure. The majority of affected children developed bilateral IH (53.3%, *n* = 16), while unilateral (right-sided) IH was found in 26.7% (*n* = 8). One patient (3.3%) suffered from incarcerated IH. In this patient, IH became clinically evident not until five months after open KPE.

**Figure 1:**
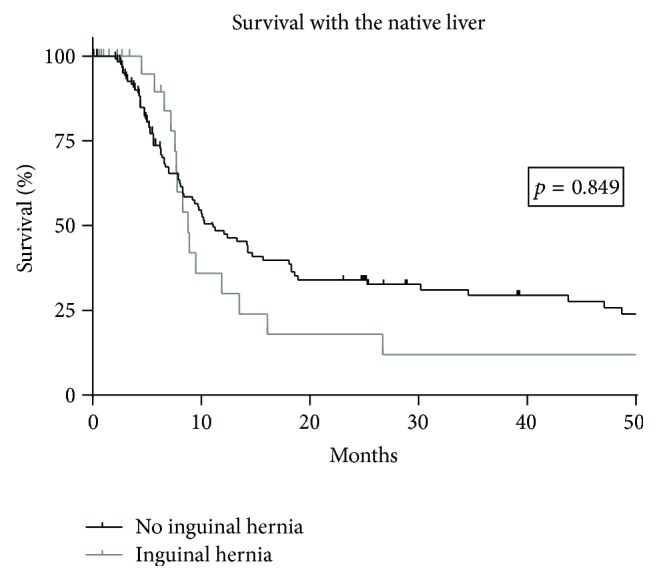
Survival with the native liver of patients with biliary atresia separated into children with inguinal hernias and nonhernia children.

In comparison to nonhernia patients, children with IH were more often males (*p* = 0.002) and suffered more often from syndromic BA (*p* = 0.028) (Table 3). In addition, hernia patients had a higher frequency of primary percutaneous drainage after KPE (*p* = 0.025). Therefore, primary drainage did not protect from IH development. Correlation analysis revealed a weak association between IH and male gender (*p* = 0.001, *r* = 0.261), syndromic BA (*p* = 0.008, *r* = 0.213), and primary drainage of the abdomen (*p* = 0.020, *r* = 0.188). Furthermore, a negative association between the development of IH and survival with the native liver could be demonstrated (*p* = 0.011, *r* = −0.204). Nevertheless, the median survival with the native liver did not differ significantly between hernia and nonhernia patients (*p* = 0.849) as illustrated in Figure 1.

Multivariate analysis revealed that male gender was an independent risk factor for the development of IH beside the syndromic form of BA and the use of primary drainage during Kasai's procedure (Table 4). Age at open KPE, gestational age, prematurity, and birth weight did not predict the development of IH in BA patients.

## 4. Discussion

Since the description of hepatoportoenterostomy by Morio Kasai in 1959, KPE constitutes the first-line therapy in patients with BA [14, 15]. However, various comorbidities and complications intimidate patients' survival with the native liver [6, 8, 9, 16, 17]. Therefore, we analyzed short-term complications developing after open KPE in order to identify potential risk factors. The results of the present study can be summarized as follows: 40.5% of patients developed moderate to severe surgical complication, IH being by far the most frequent.

Based on open processus vaginalis, indirect IH is known as the most common indication for surgery in the first months of life [18, 19]. While the incidence of IH in full-term neonates was estimated 1–5% [20–22], the first study of hernias in BA patients demonstrated an increased incidence of IH (8.1%) [11], which is corroborated by our findings revealing even a higher incidence of IH (19.6% in our BA patients). Therefore, we could confirm that BA patients suffer from a high risk for the development of IH. Referring to the literature, prematurity was described as the most common risk factor for IH in the general population conferring a 10-fold increased risk for IH development [11, 23]. In contrast, Zani and Davenport did not detect an association between prematurity and IH in BA patients [11]. Our study validated this observation. In addition, we were able to show that neither birth weight nor gestational age affected the likelihood for the development of IH in BA patients. Both factors have been described to predispose for IH development in healthy neonates [18]. Hence, we conclude that the risk profile for the development of IH differs between BA patients and the general neonatal population.

Male gender is a well-recognized risk factor for IH development in healthy children [19]. While Wilson-Storey described an overall boy : girl ratio of 4 : 1, Powell et al. found a more than 9-fold increased relative risk for IH in boys compared to female neonates [18, 22]. In BA patients, Zani and Davenport observed a higher proportion of boys in hernia patients compared to nonhernia patients [11]. Nevertheless, the authors could not detect a significant association between IH and male gender [11]. This finding is in contrast to our data clearly showing a 7-fold increased OR in case of male gender. The most likely explanation for this discrepancy is the small number of hernia patients (*n* = 10) in the study of Zani and Davenport suggesting that their study was underpowered to detect this difference [11]. Noteworthy, 30% of hernias were diagnosed prior to KPE in the Zani and Davenport study [11], while IH was diagnosed prior to KPE in only one patient in our cohort. Hence, patients' characteristics of these different groups might be diverse. With respect to the mentioned differences, Zani and Davenport did not find a correlation between IH and syndromic BA [11], while our data revealed a more than 6-fold increased risk for IH in these children. However, the high incidence of IH development after KPE may suggest that BA patients should be screened for IH before KPE, eventually facilitating hernia repair and KPE during one surgical session, thereby preventing a considerable proportion of secondary procedures in BA patients. Referring to our data, we suggest that attention should be focused particularly on children with syndromic BA.

In terms of ascites, our results validated an association between IH and primary percutaneous drainage during KPE. While 70.0% of hernia patients received primary drainage at the time of Kasai's procedure, only 46.3% of nonhernia patients were drained primarily. Thus, percutaneous ascites drainage did not prevent the development of IH in many patients. Hence, it seems questionable whether primary percutaneous drainage should be routinely performed in BA patients with ascites. However, a prospective study is needed to thoroughly address this issue.

The survival between hernia and nonhernia BA patients was similar. In particular, the incidence of complicated courses of IH due to incarcerated hernias (3.3% of our BA patients) was low which was corroborated by Zani and Davenport who did not detect incarcerated IH [11]. Therefore, it seems that IH development is not predictive of a worse outcome after KPE.

The present study has some limitations. Apart from the retrospective nature of our analysis, the follow-up time after KPE was relatively short (median 7.8 months). In addition, a substantial number of patients had to be excluded due to missing follow-up. Anyhow, 79.9% of BA patients treated over a 20-year period at our center were available for follow-up, representing the largest single-center series analyzing the incidence of IH in BA patients to date.

## 5. Conclusions

BA patients are at increased risk for the development of IH in the short-term course after KPE. Although liver dysfunction and ascites may predispose for the development of IH, primary percutaneous drainage did not prevent IH development in most patients. In addition, the presented study suggests that male gender and syndromic BA are associated with an increased risk for the development of IH. Further prospective and multicenter studies are required to determine risk factors for IH in these children and to evaluate the impact of a prophylactic inspection of the inguinal canal in order to prevent the most frequent complication and additional surgical procedures in BA patients.

## Figures and Tables

**Table 1:** Frequency of congenital anomalies in biliary atresia patients according to syndromic manifestation of the disease.

Congenital anomalies	Syndromic BA (%)	Nonsyndromic BA (%)	*P* value
Number of patients (%)	7 (4.6%)	146 (95.4%)	—
Cardiovascular anomaly	**6** (**85.7**%)	**23** (**15.8**%)	**<0.001**
Gastrointestinal anomaly	**4** (**57.1**%)	**10** (**6.8**%)	**0.001**
Genitourinary anomaly	**2** (**28.6**%)	**2** (**1.4**%)	**0.010**
Pulmonary anomaly	1 (14.3%)	1 (0.7%)	0.090
Situs inversus	**2** (**28.6**%)	—	**0.002**
Splenic anomaly	**4** (**57.1**%)	—	**<0.001**

**Table 2:** Frequency of surgical complications observed in biliary atresia patients after open Kasai's hepatoportoenterostomy including the proportion of lethal complications.

List of complications	Number of cases (%)	Lethal cases (%)
Inguinal hernia	30 (40.0%)	—
Hemorrhage of esophageal varices	11 (14.7%)	1 (9.1%)
Anastomotic leak of bile duct	5 (6.7%)	—
Incisional hernia	5 (6.7%)	—
Intestinal obstruction	5 (6.7%)	1 (20.0%)
Cholangitis with need for central venous catheter	4 (5.3%)	—
Postoperative hemorrhage	4 (5.3%)	—
Anastomotic leak of foot-point anastomosis	2 (2.7%)	—
Biloma	2 (2.7%)	—
Umbilical hernia	2 (2.7%)	—
Abscess surgical area	1 (1.3%)	—
Intestinal hemorrhage	1 (1.3%)	—
Intestinal ischemia	1 (1.3%)	1 (100%)
Perforation of the ileum	1 (1.3%)	—
Splenectomy due to hypersplenism	1 (1.3%)	—

**Table 3:** Study population and patients' characteristics according to the appearance of inguinal hernias in children with biliary atresia.

Patients' characteristics	All patients	Inguinal hernia	No inguinal hernia	*P* value
Number of patients (%)	153 (100%)	30 (19.6%)	123 (80.4%)	—
Age at KPE <60 days (%)	89 (58.2%)	19 (63.3%)	70 (56.9%)	0.545
Intraoperative drainage (%)	**78** (**51.0**%)	**21** (**70.0**%)	**57** (**46.3**%)	**0.025**
Male gender (%)	**67 **(**43.8**%)	**21** (**70.0**%)	**46** (**37.4**%)	**0.002**
Premature birth (%)	23 (15.0%)	7 (23.3%)	16 (13.0%)	0.163
Syndromic BA (%)	**7** (**4.6**%)	**4** (**13.3**%)	**3** (**2.4%**)	**0.028**

**Table 4:** Multivariate regression analysis referring to inguinal hernias analyzing patients' characteristics as potential predictors (Nagelkerke *R* ^2^ = 0.317).

Predictor	Regression coefficient	Odds ratio (OR)	95% confidence interval (95% CI)	*P* value
Age at KPE <60 days	−0.119	0.888	0.315–2.499	0.821
Gestational age	−0.048	0.953	0.696–1.306	0.766
Intraoperative drainage	**1.788**	**5.975**	**1.966**–**18.156**	**0.002**
Male gender	**1.991**	**7.319**	**2.066**–**25.928**	**0.002**
Premature birth	1.797	6.030	0.841–43.242	0.074
Syndromic BA	**1.934**	**6.918**	**1.110**–**43.112**	**0.038**
Weight	0.000	1.000	0.999–1.001	0.912
Constant	−1.938	0.144	—	0.749

## List of Abbreviations

BA: Biliary atresia
IH: Inguinal hernia
IQR: Interquartile range
KPE: Kasai's hepatoportoenterostomy
LTx: Liver transplantation
OR: Odds ratio
95% CI: 95% confidence interval.
